# Primary Care by Telehealth and Care Quality in the Veterans Health Administration

**DOI:** 10.1001/jamanetworkopen.2025.59940

**Published:** 2026-02-17

**Authors:** Jonathan Staloff, Eric Gunnink, Jorge Rojas, Edwin S. Wong, Jacqueline M. Ferguson, Donna M. Zulman, Karin Nelson, Ashok Reddy

**Affiliations:** 1 Center of Innovation for Veteran-Centered and Value-Driven Care, VA Puget Sound Health Care System, Seattle, Washington; 2 Department of Family Medicine, University of Washington, Seattle; 3 Department of Health Systems and Population Health, University of Washington School of Public Health, Seattle; 4 Center for Innovation to Implementation in the VA Palo Alto Health Care System, Palo Alto, California; 5 Department of Medicine (Primary Care and Population Health), Stanford University, Stanford, California; 6 Department of Medicine, University of Washington, Seattle

## Abstract

**Importance:**

As telehealth (ie, telephone and video) becomes a larger component of primary care, understanding its impact on care quality is critical.

**Objective:**

To evaluate whether the proportion of primary care received via telehealth is associated with differences in quality-of-care outcomes among veterans who frequently use primary care.

**Design, Setting, and Participants:**

This is a retrospective cohort study of veterans empaneled to Veterans Health Administration (VHA) primary care in fiscal years 2022 and 2023 (October 1, 2021, to September 30, 2023) with 3 or more primary care visits. Telehealth proportion categories were none (0.0% primary care visits telehealth), low (>0.0% to <28.6%), intermediate (28.6% to <50.0%), or high (≥50.0%).

**Exposure:**

Proportion of primary care delivered via telehealth.

**Main Outcomes and Measures:**

The primary outcomes were influenza vaccination, hypertension control, statin therapy and adherence, and screenings and/or counseling for depression, tobacco, and alcohol use. Multivariable logistic regression was used to estimate adjusted average marginal effects (AMEs), controlling for sociodemographic, geographic, and clinical characteristics.

**Results:**

This study included 744 599 veterans (mean [SD] age, 65 [15] years; 638 289 male [86%]). Compared with veterans receiving in-person care only, those who received a low proportion of care via telehealth had similar quality of outcomes for all cardiovascular and behavioral health measures. Influenza vaccination rates were modestly lower in the low-telehealth group vs the in-person only group (age ≥66 years, AME, −1.93% [95% CI, −2.58% to −1.29%]; age 19-65 years, AME, −1.57% [95% CI, −2.28% to −0.86%]). High telehealth users (≥50% telehealth) had the lowest adjusted likelihoods for most quality outcomes, including influenza vaccination (age ≥66 years, AME, −8.96% [95% CI, −9.84% to −8.07%]; age 19-65 years, AME, −9.72% [95% CI, −10.84% to −8.60%]) statin adherence (AME, –2.03% [95% CI −2.93% to −1.14%]) and depression screening (AME, –2.14% [95% CI, −3.20% to −1.08%]).

**Conclusions and Relevance:**

In this cohort study of veterans with 3 or more primary care visits, primary care quality was similar for individuals who received all in-person care and those receiving low or intermediate proportions of telehealth. However, high telehealth use was associated with lower quality for several services, especially those requiring in-person interaction. Findings demonstrate the viability of hybrid telehealth and in-person models. Additional resources might be needed to ensure high-quality primary care for high proportion telehealth users.

## Key Points

**Question:**

Is the proportion of primary care delivered via telehealth in the Veterans Health Administration associated with differences in quality?

**Findings:**

In this cohort study of 744 599 veterans, quality was the same between low proportion telehealth users and nontelehealth users for most measures. Veterans with a high proportion of telehealth use had lower influenza vaccination receipt and cardiovascular and behavioral health measure performance compared with nontelehealth users.

**Meaning:**

These findings suggest that a low proportion of telehealth use maintains primary care quality comparable to that of in-person care; however, a high proportion of telehealth use was associated with lower quality, especially for services needing in-person care (eg, vaccines).

## Teaser

This cohort study evaluates whether the proportion of primary care received via telehealth (telephone and video) is associated with differences in quality-of-care outcomes among Veterans Health Administration (VHA) patients who frequently use primary care.

## Introduction

The Veterans Health Administration (VHA) continues to use telehealth (telephone and video visits) to deliver primary care services.^1^ As telehealth takes a larger role in primary care delivery, it is critical to understand the potential impacts telehealth has on quality.

Some prior studies assessed the quality of individual primary care visits by care modality.^2,3,4,5,6^ Others explored broader differences in primary care quality between patients who receive any telehealth or in-person care only.^7,8^ One study^9^ considered whether care quality varies depending on how extensively a health system uses telehealth relative to others. Yet, whether the proportion of an individual patient’s primary care delivered via telehealth affects quality remains understudied.

To address this evidence gap, our objective was to determine whether the proportion of primary care received via telehealth is associated with differences in quality of care among frequent primary care users (≥3 primary care evaluation and management visits). Specifically, we examined the degree to which the proportion of primary care received via telehealth was associated with differences in national quality measures for influenza immunization delivery, cardiovascular disease care, and behavioral health screenings and counseling.

## Methods

### Study Design

We conducted a retrospective cohort analysis of a national sample of veterans empaneled to VHA primary care in fiscal years (FYs) 2022 and 2023 (October 1, 2021, to September 30, 2023). We restricted the cohort to veterans with 3 or more primary care encounters in FY 2022 to ensure a meaningful assessment of a population with substantial primary care continuity and exposure to primary care preventive and chronic disease care processes (eAppendix 1 in Supplement 1).^10^ This approach aligns with prior VHA quality-of-care studies using visit-based denominators to minimize misclassification among infrequent users.^11^ Additionally, because our exposure was defined as the proportion of visits conducted via telehealth, this approach allowed for a more stable proportion telehealth measure. For example, veterans with only 1 or 2 visits could have telehealth proportions of 0% or 100%, which may yield unstable estimates and limited interpretive value. We defined our exposure as a 4-level categorical variable of the proportion of primary care evaluation and management visits that veterans receive via telehealth (sum of video and phone visits, using definitions for telehealth outlined in prior work).^1^ Then, we compared receipt of recommended care on primary care relevant quality measures, by exposure group. We conducted multivariable logistic regression to compare probability differences in receiving recommended care between groups, adjusted for covariates. This work was carried out as a quality improvement evaluation and designated nonresearch under the authority of the VHA Office of Primary Care, and, thus, was exempt from institutional review board review and the need for informed consent. This article follows Strengthening the Reporting of Observational Studies in Epidemiology (STROBE) reporting guidelines.^12^

### Data Source and Participants

Our data sources included the VHA Support Service Center Capital Assets Databases (a web-based project application and tracking database)^13^ and the Primary Care Management Module (an enterprise application used to track health care teams and patient and team member relationships).^14^ We included a national sample of veterans empaneled to VHA primary care in FY 2022 to 2023 who had 3 or more evaluation and management visits of any modality (in-person, video, phone) in FY 2022. We obtained patient and clinician characteristics, and patient encounter data from the VHA Corporate Data Warehouse, a repository for Veterans’ electronic health care record data.^15^

### Primary Variables

#### Exposure Variable or Intervention

The exposure was a categorical variable measuring the proportion of total annual primary care evaluation and management visits conducted via telehealth. First, using data in FY 2022 (October 1, 2021, to September 30, 2022), we identified primary care evaluation and management visits and their modality (in-person, telephone, and video) using VHA Managerial Cost Accounting specific stop codes (eAppendix 1 in Supplement 1).^1,16^ Stop codes are VA identifiers that identify the type of clinical services provided at a given clinic location (eg, primary care, cardiology, or pulmonology). Using identified primary care evaluation and management visits, we calculated the proportion that were delivered via telehealth as follows: telehealth / (telehealth + in-person). We identified veterans with a primary care telehealth proportion of 0.0%. Of the remaining veterans, the proportion of telehealth use was described as mean (39.0%), 25th percentile (28.6%), median (33.0%), and 75th percentile (50.0%). On the basis of these data, we discussed as a team a categorization with face validity based on clinical experience, and that would allow comparisons of various telehealth proportions while also including telehealth nonusers in our study. We subsequently generated a 4-level categorical variable based on tertiles of the distribution of telehealth proportion above zero: no telehealth, low telehealth (>0.0% to <28.6%), intermediate telehealth (≥28.6% to <50.0%), and high telehealth (≥50.0%). We compared mean total number of primary care visits among the exposure groups using 1-way analysis of variance.

#### Primary Care Outcome Measures

We selected outcome measures in FY 2023 (October 1, 2022, to September 30, 2023). Outcome measures were selected from 2 sources, the VHA’s electronic quality measure set (similar to the Healthcare Effectiveness Data and Information Set) and from the external peer review program, a VHA quality assurance initiative that involves external peer review and performance abstraction from a randomized sample of individual patient records.^17,18^ We selected measures that reflect care of physical and behavioral health care conditions commonly seen in primary care, and may be influenced by visit care modality. Our selected measures include those related to delivery of preventive services and chronic disease management. We only included quality measures with a 1-year lookback period, since FY 2023 performance on outcomes with a longer lookback period (eg, colon cancer screening, with a 10-year lookback) could be based on services provided prior to our exposure. Thus, our selected quality measures encompassed vaccine administration (annual influenza vaccine administration, stratified as 1 measure for veterans aged 19-65 years and 1 for those aged ≥66 years), cardiovascular care (hypertension control, statin therapy, and statin adherence for veterans with cardiovascular disease), and behavioral health care screenings and counseling (tobacco use screening, tobacco cessation counseling, alcohol misuse screening, and depression screening) (eAppendix 2 in Supplement 1).^19^

#### Covariates

Patient demographic characteristics were abstracted from FY 2022 and included age (years); sex (male or female); marital status (married or not married); and race and ethnicity. Race and ethnicity were included as covariates to address potential concerns of confounding related to this construct. Specifically, prior studies identified the potential for quality of care differences by race, and others identified race and ethnicity as a variable associated with differences in telehealth use.^20,21,22^ Race and ethnicity were identified with an algorithm that uses self-reported race and ethnicity when available, and otherwise uses administrative databases.^23^ Categories included Hispanic, non-Hispanic Black, non-Hispanic White, and other (which included those originally identified as other race as well as those originally identified as American Indian or Alaska Native, Asian, multiracial, Native Hawaiian, and Pacific Islander). Area-level characteristics included urban vs rural address (defined using the VHA Urban-Rural-Highly-Rural classification system, which we condensed to urban and rural)^24^; driving distance (miles) to the nearest VHA primary care site, neighborhood socioeconomic status index decile (range 0-9, with higher decile reflecting higher neighborhood level socioeconomic status), and Federal Communications Commission broadband availability.^25,26^ Broadband availability was classified as inadequate (download speed ≤25 MB/s and upload speed ≤3 MB/s), adequate (download speed ≥25 and <100 MB/s and upload speed ≥5 and <100 MB/s), or optimal (download and upload speeds ≥100 MB/s) based on data reported at the Census block by internet providers.^25^ Additional clinical patient characteristics included those focused on clinical comorbidity and utilization, including JEN Frailty Index,^27^ Gagne index,^28,29^ and total number of primary care evaluation and management visits.

### Statistical Analysis

In unadjusted analysis, we used a χ^2^ equality of proportions without continuity correction to compare unadjusted differences in receipt of recommended primary care by proportion of primary care received by telehealth. In adjusted analyses, we conducted multivariable logistic regressions to estimate the association between proportion telehealth and quality outcomes, adjusting for covariates. Using the method of recycled predictions, we calculated average marginal effects (AMEs), which communicated the difference in the probability of meeting a given quality criteria between exposure groups.^30^ Additionally, since our analyses were exploratory rather than testing a priori hypotheses, and since each model outcome analyzed different subcohorts (eg, veterans with hypertension) rather than a consistent cohort, we did not correct for multiple hypothesis testing.^31^

We calculated variance inflation factors to assess multicollinearity among the variables. No variables were removed as the variance inflation factor was less than 5 for all variables, below the threshold of 10, which is considered an indicator of multicollinearity being present.^32^ We addressed covariate missingness using multiple imputation using the mice package in R, generating 20 imputed datasets, and reported results from pooled models. All adjusted models accounted for facility-level clustering using robust SEs.^33,34^ All analyses were performed using R statistical software version 4.5.1 (R Project for Statistical Computing). The threshold for statistical significance was *P* < .05 or the 95% CI for marginal effects estimates including 0.00.

## Results

There were 744 599 veterans with 3 or more FY 2022 primary care visits in the cohort, accounting for 11.6% of all primary care empaneled veterans during the study period. The mean (SD) age was 65 (15) years; 638 289 (86%) were male, and 106 310 (14%) were female. Reported race and ethnicity was 73 571 (10%) Hispanic veterans, 158 703 (22%) non-Hispanic Black veterans, 462 561 (63%) non-Hispanic White veterans, and 34 421 veterans (5%) of other races. The mean (SD) number of annual in-person visits was 4.04 (2.21) in the no telehealth group (364 365 veterans [48.9%]); 4.30 (2.27) in-person, 0.17 (0.43) telephone, and 0.97 (0.53) video visits in the low telehealth group (95 002 veterans [12.8%]); 2.69 (1.35) in-person, 0.19 (0.61) telephone, and 1.34 (1.01) video visits in the intermediate group (164 827 veterans [22.1%]); and 2.06 (1.18) in-person, 1.11 (1.87) telephone, and 1.64 (1.27) video visits in the high telehealth group (120 125 veterans [16.1%]). The mean (SD) total number of primary care visits differed across groups (no telehealth, 4.04 [2.21] visits; low telehealth, 5.43 [2.48] visits; intermediate telehealth, 4.22 [2.32] visits; and high telehealth, 4.81 [2.32] visits). High telehealth users were younger (aged 62 vs 68 years), more likely to be female (19 130 veterans [16%] vs 41 136 veterans [11%]), less likely to be non-Hispanic White (70 676 veterans [60%] vs 238 937 veterans [67%]), more likely to be urban residing (82 181 veterans [69%] vs 222 340 veterans [61%]), had fewer comorbidities (97 360 veterans [81%] vs 271 520 veterans [74%] with Gagne score <1), and had lower degrees of frailty (26 207 veterans [22%] vs 100 138 veterans [28%] in the highest quartile of JEN frailty index) compared with no telehealth users (Table 1).

**Table 1.** Characteristics of Veteran Primary Care Users, by Telehealth Utilization Category

Characteristic	Veterans, No. (%)				*P* value
	No telehealth (n = 364 645)	Low telehealth, <28.6% (n = 95 002)	Intermediate telehealth, ≥28.6% and <50.0% (n = 164 827)	High telehealth, ≥50.0% (n = 120 125)	
Age, y					
18-34	10 226 (3)	5026 (5)	11 024 (7)	7426 (6)	<.001
35-44	22 251 (6)	9665 (10)	21 622 (13)	15 976 (13)	
45-54	30 000 (8)	12 114 (13)	23 660 (14)	17 065 (14)	
55-64	61 410 (17)	19 186 (20)	32 812 (20)	22 187 (18)	
65-74	102 590 (28)	23 759 (25)	36 161 (22)	25 834 (22)	
≥75	138 166 (38)	25 252 (27)	39 548 (24)	31 637 (26)	
Sex					
Female	41 136 (11)	17 195 (18)	28 849 (18)	19 130 (16)	<.001
Male	323 509 (89)	77 807 (82)	135 978 (82)	100 995 (84)	
Race and ethnicity					
Hispanic	33 286 (9)	10 230 (11)	17 570 (11)	12 485 (11)	<.001
Non-Hispanic Black	70 876 (20)	22 162 (24)	37 606 (23)	28 059 (24)	
Non-Hispanic White	238 937 (67)	55 666 (60)	97 282 (60)	70 676 (60)	
Other^a^	14 859 (4)	4975 (5)	8435 (5)	6152 (5)	
Neighborhood socioeconomic status, decile^b^					
0-3	151 390 (43)	37 022 (41)	59 740 (39)	45 461 (40)	<.001
4-6	120 699 (34)	31 143 (34)	53 977 (35)	39 321 (35)	
7-9	78 349 (22)	22 236 (25)	40 826 (26)	28 975 (25)	
Marital status					
Married	189 733 (52)	48 366 (51)	87 935 (53)	64 612 (54)	<.001
Unmarried	174 912 (48)	46 636 (49)	76 892 (47)	55 513 (46)	
Urban-rural status					
Urban	222 340 (61)	64 463 (68)	111 275 (68)	82 181 (69)	<.001
Rural	139 730 (39)	29 686 (32)	52 003 (32)	36 823 (31)	
Gagne score					
<1	271 520 (74)	74 006 (78)	136 315 (83)	97 360 (81)	<.001
1	40 264 (11)	9327 (10)	13 570 (8.2)	10 111 (8)	
2	22 857 (6)	5089 (5)	6697 (4)	5348 (5)	
≥3	30 004 (8)	6580 (7)	8245 (5.0)	7306 (6)	
Driving distance to primary care, miles					
≤5 (quartile 1)	105 778 (29)	27 693 (29)	43 309 (27)	30 439 (26)	<.001
6-10 (quartile 2)	82 385 (23)	23 903 (25)	40 899 (25)	29 749 (25)	
11-19 (quartile 3)	79 928 (22)	21 903 (23)	40 117 (25)	30 403 (26)	
≥20 (quartile 4)	94 119 (26)	20 692 (22)	39 003 (24)	28 419 (24)	
No. of in-person primary care visits, mean (SD)	4.04 (2.21)	4.30 (2.27)	2.69 (1.35)	2.05 (1.18)	<.001
No. of primary care telephone visits, mean (SD)	0	0.17 (0.43)	0.19 (0.61)	1.11 (1.87)	<.001
No. of primary care video visits, mean (SD)	0	0.97 (0.53)	1.34 (1.01)	1.64 (1.27)	<.001
JEN Frailty Index					
Quartile 1	130 088 (36)	32 602 (34)	75 649 (46)	53 143 (44)	<.001
Quartile 2	72 301 (20)	19 326 (20)	32 769 (20)	23 170 (19)	
Quartile 3	61 491 (17)	16 601 (18)	24 036 (15)	17 383 (14)	
Quartile 4	100 138 (28)	26 302 (28)	32 057 (19)	26 207 (22)	
Internet speed adequacy					
Adequate	199 847 (56)	49 889 (54)	83 612 (53)	61 803 (53)	<.001
Inadequate	29 687 (8)	5952 (6)	10 385 (7)	8183 (7)	
Optimal	127 687 (36)	36 472 (40)	65 025 (41)	46 605 (40)	

^a^ Racial and ethnic groups categorized as other includes those originally identified as other as well as those who identified as American Indian or Alaska Native, Asian, multiracial, Native Hawaiian, and Pacific Islander.

^b^ Higher deciles reflect higher socioeconomic status.

### Unadjusted Analysis

The largest observed difference between groups was for the influenza vaccine measure. The no telehealth group met the measure most frequently (age 19-65 years, 147 160 veterans [50.0%]; age ≥66 years, 220 138 veterans [71.5%]), and the high telehealth (age 19-65 years, 68 485 veterans [37.9%]; age ≥66 years, 50 403 veterans [62.8%]) group the least frequently (Table 2). For the hypertension control and statin therapy measures, there was a small but statistically significant difference between groups, wherein the low telehealth group met the measures most frequently and the high telehealth the least frequently. There was no statistically significant difference between groups in tobacco cessation counseling. For all other measures, there was a small statistically significant difference between groups with an incremental response, wherein the no telehealth group met the measures most frequently and the high telehealth group the least frequently (Table 2).

**Table 2.** Unadjusted Proportion of Individuals Meeting Quality Measure, Stratified by Proportion Primary Care Telehealth Use

Quality measure	Percentage meeting measure (No. eligible for measure)				*P* value, χ^2^
	No telehealth	Low telehealth	Intermediate telehealth	High telehealth	
Influenza immunization					
Age 19-65 y	50.0 (147 160)	48.6 (51 963)	42.5 (98 046)	37.9 (68 485)	<.001
Age ≥66 y	71.5 (220 138)	70.2 (43 689)	66.5 (67 140)	62.8 (50 403)	<.001
Cardiovascular care					
Hypertension control	75.8 (207 843)	76.1 (50 898)	74.7 (80 955)	73.2 (57 296)	<.001
Statin therapy for CVD	91.9 (32 224)	92.3 (7398)	90.9 (10 884)	90.2 (8475)	<.001
Statin adherence for CVD	85.9 (28 712)	85.1 (6615)	85.0 (9439)	83.6 (7306)	<.001
Behavioral health measures					
Annual depression screening	95.5 (12 340)	95.2 (2982)	94.3 (4401)	93.0 (2896)	<.001
Annual tobacco use screening	95.5 (21 629)	94.9 (6131)	94.0 (8668)	92.8 (5719)	<.001
Tobacco cessation counseling	99.1 (4218)	99.4 (1078)	99.5 (1521)	99.4 (1043)	.40
Annual alcohol misuse screening	96.2 (21 647)	95.7 (6146)	94.9 (8690)	94.0 (5718)	<.001

Abbreviation: CVD, cardiovascular disease.

### Multivariable Regression Analyses

#### Influenza Vaccination

We observed an incremental response, wherein veterans had a lower likelihood of meeting the measure as their proportion of primary care telehealth increased. In the 19- to 65-year-old subgroup, compared with the no telehealth subgroup, veterans classified as low (−1.57%; 95% CI, −2.28% to −0.86%), intermediate (−4.92%; 95% CI, −5.63% to −4.20%), and high (−9.72%; 95% CI, −10.84% to −8.60%) telehealth users had lower probability of influenza vaccination. We observed a similar association among those aged 66 years and older, where low telehealth users had an AME of −1.93% (95% CI, −2.58% to −1.29%), intermediate users had an AME of −4.68% (95% CI, −5.30% to −4.06%), and high telehealth users had an AME of −8.96% (95% CI, −9.84% to −8.07%) (Figure and Table 3).

**Figure.**
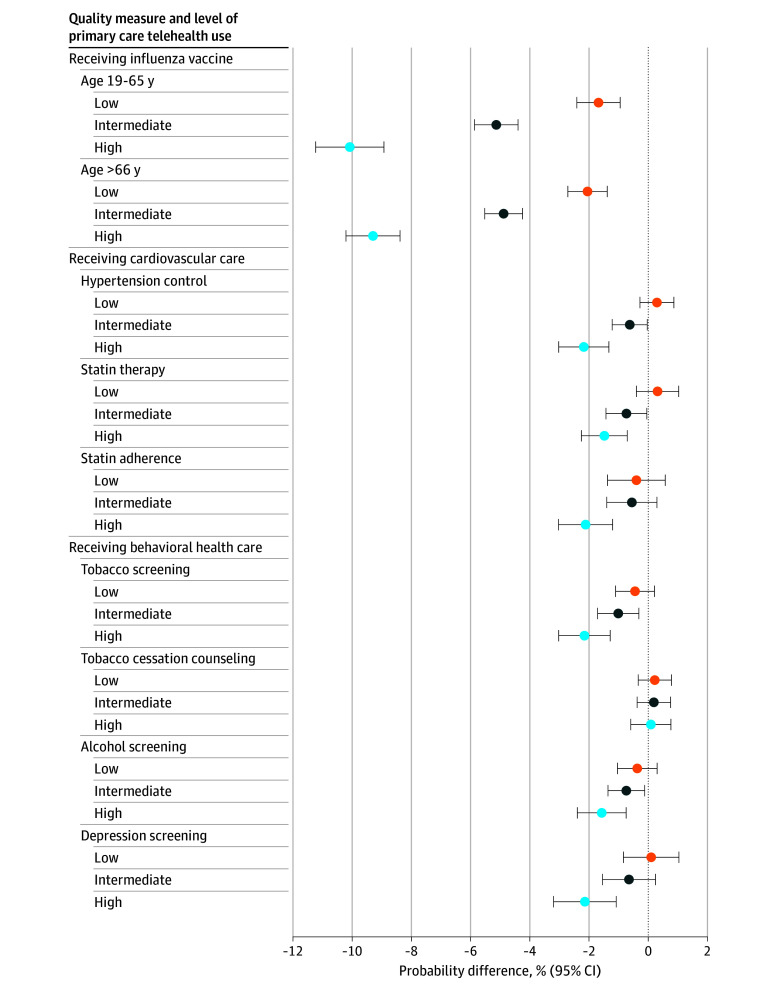
Adjusted Average Marginal Effects Probability Differences of Meeting Quality Measure by Proportion Primary Care Telehealth Use

**Table 3.** Adjusted Average Marginal Effects Probability Differences of Meeting Quality Measure by Proportion Primary Care Telehealth Use

Quality outcome	No. of veterans	Average marginal effect, % (95% CI)^a^		
		Low telehealth	Intermediate telehealth	High telehealth
Influenza vaccination				
Age 19-65 y	363 038	−1.57 (−2.28 to −0.86)	−4.92 (−5.63 to −4.20)	−9.72 (−10.84 to −8.60)
Age ≥66 y	377 996	−1.93 (−2.58 to −1.29)	−4.68 (−5.30 to −4.06)	−8.96 (−9.84 to −8.07)
Hypertension control	394 106	0.33 (−0.26 to 0.92)	−0.57 (−1.15 to 0.01)	−2.09 (−2.92 to −1.26)
Statin therapy	58 564	0.35 (−0.35 to 1.05)	−0.68 (−1.35 to 0.00)	−1.41 (−2.17 to −0.65)
Statin adherence	51 708	−0.35 (−1.30 to 0.61)	−0.50 (−1.33 to 0.33)	−2.03 (−2.93 to −1.14)
Tobacco screening	41 793	−0.42 (−1.07 to 0.23)	−0.98 (−1.67 to −0.29)	−2.11 (−2.97 to −1.25)
Tobacco cessation counseling	7797	0.24 (−0.31 to 0.80)	0.21 (−0.35 to 0.77)	0.11 (−0.56 to 0.78)
Alcohol screening	41 845	−0.37 (−1.04 to 0.30)	−0.74 (−1.36 to −0.12)	−1.57 (−2.39 to −0.74)
Depression screening	22 412	0.10 (−0.83 to 1.04)	−0.65 (−1.55 to 0.24)	−2.14 (−3.20 to −1.08)

^a^ The reference group is the no telehealth group.

#### Cardiovascular Care and Behavioral Health Care

Only the high telehealth group had a lower likelihood of meeting the hypertension control (AME, −2.09%; 95% CI, −2.92% to −1.26%), statin therapy (AME, −1.41%; 95% CI, −2.17% to −0.65%), and statin adherence (AME, −2.03%; 95% CI, −2.93% to −1.14%) measures than the no telehealth group (Figure and Table 3). Only the high telehealth group had a lower likelihood of receiving annual depression screening compared with the no telehealth group (AME, −2.14%; 95% CI, −3.20% to −1.08%) (Figure and Table 3). Both the intermediate (AME, −0.98%; 95% CI, −1.67% to −0.29%) and high (AME, −2.11%; 95% CI, −2.97% to −1.25%) telehealth groups had lower likelihood of receiving tobacco use screening, but there was no difference across all groups in receipt of tobacco cessation counseling compared with the reference group of no telehealth. The intermediate (AME, −0.74%; 95% CI, −1.36% to −0.12%) and high (AME, −1.57%; 95% CI, −2.39% to −0.74%) telehealth groups also had slightly lower proportion likelihood of receiving annual alcohol use screening. There was no difference between the low telehealth and no telehealth groups for all behavioral health measures (Figure and Table 3).

## Discussion

In this national cohort study of frequent primary care users, veterans with a low proportion of telehealth visits received the same quality of care as telehealth nonusers for all measures except for influenza vaccination. Among veterans with an intermediate proportion of telehealth visits, quality of care was equivalent to that for telehealth nonusers for some measures (hypertension control, statin adherence and therapy, tobacco cessation counseling, and depression screening), slightly lower for others (tobacco screening and alcohol screening), and substantially lower only for influenza vaccination. Collectively, these findings suggest that quality preventive services and chronic disease management can be delivered via many mixtures of in-person and telehealth use.

However, with the exception of tobacco cessation counseling, our study also demonstrated a consistent decrease in care quality when telehealth makes up one-half or more of encounters. The magnitude of this association was most pronounced for influenza vaccines, which differs from others in this study since it requires in-person care. Therefore, influenza vaccine administration and other processes that require in-person care (eg, diabetic retinopathy screen) could be overlooked for patients receiving higher proportions of telehealth. Thus, additional workflows may be needed to ensure that high telehealth users come to VHA or community clinics in-person to receive such services.

This study provides new evidence about the role of telehealth in primary care. Prior related studies have mostly focused on comparing patients who have received any telehealth vs those who have exclusively used in-person visits, rather than examining telehealth as a graded exposure. We identified only 1 study that adjusted for overall health care utilization,^7^ which is important for contextualizing telehealth utilization as part of overall quantity of care. Furthermore, results from prior literature have been mixed. For example, 1 study demonstrated no difference in hemoglobin A_1c_, hypertension, or low-density lipoprotein control between patients who have used any telehealth for primary care and those who have not.^7^ In contrast, another study found that patients exposed to telehealth across ambulatory care settings were more likely to receive some recommended chronic disease–related testing (eg, lipid panels for cardiovascular disease), but less likely to receive some chronic disease–related medications (eg, antiplatelets for cardiovascular disease).^8^ A separate study found that higher amounts of telehealth exposure were associated with increased adherence to metformin and statins. However, that study examined telehealth utilization at the system rather than patient level or within primary care contexts specifically.^9^

Our study is the first, to our knowledge, that examines how the proportion of primary care received via telehealth—as a graded exposure—is associated with care quality in a nationally integrated system. This analysis is important considering the mixed findings in literature that examined telehealth as a binary exposure. Additionally, our study specifically focuses on regular users of primary care, thus offering insights into how telehealth can complement or substitute for in-person primary care.

Overall, our study demonstrates that low and intermediate proportions of telehealth provide equivalent or near-equivalent quality as in-person only primary care. Complementing prior literature demonstrating that telehealth improves access to primary care, our study supports telehealth as a component to patients’ receipt of primary care services.^35,36,37^ However, improved access to primary care via telehealth should be balanced by monitoring of care quality. Our study highlights that at high proportions of telehealth use, key measures of primary care quality may be lower without an in-person encounter.

Our work can inform ongoing policy discussions outside the VHA, including questions about long-term reimbursement for telehealth in Medicare and Medicaid.^38,39^ Specifically, since prior studies have demonstrated a high degree of comparability between VHA and Medicare populations, our study adds to the evidence supporting long-term reimbursement and the importance of telehealth for frequent primary care users.^40^ Indeed, more than one-half of veterans in this study used at least some telehealth for their primary care. Moreover, these findings suggest that telehealth can play an important role in quality primary care delivery, adding to prior literature demonstrating telehealth’s role in primary care access.^41,42,43^ Ultimately, this study provides supporting evidence for telehealth as a viable modality for quality primary care.

### Limitations

This study has limitations. First, our study investigates the association between utilization in FY 2022 with outcomes in FY 2023, a time during which utilization may change. However, since we cannot isolate which visits came before when a particular measure was assessed within each year, our use of FY as a separator allowed us to ensure that exposure preceded outcomes. Second, because this study examined telehealth proportion, we focused on veterans with 3 or more evaluation and management visits to ensure stability in the exposure variable. Although findings do not generalize to individuals with fewer than 3 primary care visits, they will be applicable to patients who have high continuity with primary care.^44,45^ Nonetheless, this design choice, while improving measurement stability, could exclude less-engaged veterans who may experience different care patterns. Therefore, results should be interpreted as applying primarily to primary care users with high continuity.

Third, the focus of our study was specifically on the quality of VHA primary care delivery and does not necessarily capture care quality outside VHA for every measure. However, that this population had many VHA primary care visits suggests that they likely received most of their primary care from VHA rather than other sources. Fourth, it is possible that the VHA incompletely captured influenza vaccines delivered in outside systems. However, we expect this limitation would be mitigated by the fact that the VHA began querying immunization data from state or jurisdiction immunization information systems via the Center for Disease Control and Prevention’s Immunization Gateway in June 2023. This automated process increased the VHA’s ability to capture vaccine administration outside by non-VHA institutions, even though with these data it is likely that our capture of influenza vaccines delivered in the community is incomplete.^35^ Fifth, although we selected measures spanning multiple domains of primary care quality, there are other elements of primary care quality not captured in this analysis (eg, convenience or access) that influence telehealth utilization. Sixth, our approach included only physician (MD or DO), nurse practitioner, or physician assistant visits for the exposure. It did not include a comprehensive understanding of all potential encounters with the primary care team (eg, pharmacists or nurses). Seventh, while our regression approach adjusted for a comprehensive set of patient, clinical, and contextual covariates, it assumes no unmeasured confounding. However, unmeasured factors such as veterans’ care preferences, digital literacy, use of non-VHA care, and other factors could affect both their likelihood of using telehealth and their observed quality of care. These unmeasured characteristics may partly explain observed associations, particularly for outcomes requiring in-person interaction (eg, influenza vaccination). Therefore, results should be interpreted as associations rather than causal effects.

## Conclusions

In this cohort study of veterans with 3 or more evaluation and management primary care visits during the 1-year study period, we found that a low proportion of primary care via telehealth offered equivalent quality across most outcomes, and differences in care quality between intermediate telehealth and no telehealth groups were often small or not statistically significant. The findings also suggest that regular users of VHA primary care who receive a majority share of visits via telehealth are much less likely to receive the influenza vaccine than no, low, and intermediate telehealth users. Therefore, clinicians and health system leaders should develop additional proactive care processes to either bring high-proportion telehealth patients in-person to ensure flu vaccine administration, or facilitate influenza vaccination outside the clinic (eg, mobile health clinics or community partnerships).^36,37^ Finally, a smaller but consistent difference was observed in other care quality measures between high telehealth and no telehealth veterans. This finding suggests that additional resources might be needed to ensure high-quality primary care for high proportion telehealth users, and that improvements in access to primary care that may come at high telehealth proportions should be balanced by close monitoring of care quality.

## Notes

Supplement 1. eAppendix 1. Method of Abstracting Primary Care Evaluation and Management Visits eAppendix 2. Measure Names and Definitions

Supplement 2. Data Sharing Statement
