# Supplementary material for: Primary Care by Telehealth and Care Quality in the Veterans Health Administration
Source: JAMA Netw Open. 2026 Feb 17;9(2):e2559940. doi: 10.1001/jamanetworkopen.2025.59940 (PMC12914489; doi:10.1001/jamanetworkopen.2025.59940)
Supplement: Supplement 1. — eAppendix 1. Method of Abstracting Primary Care Evaluation and Management Visits eAppendix 2. Measure Names and Definitions [file jamanetwopen-e2559940-s001.pdf]

Supplemental Online Content

Staloff J, Gunnink E, Rojas J Jr, et al. Primary care by telehealth and care quality in the Veterans Health Administration. *JAMA Netw Open*. 2026;9(2):e2559940.
doi:10.1001/jamanetworkopen.2025.59940

eAppendix 1. Method of Abstracting Primary Care Evaluation and Management Visits
eAppendix 2. Measure Names and Definitions

This supplemental material has been provided by the authors to give readers additional information about their work.

© 2026 Staloff J et al. *JAMA Network Open*.

eAppendix 1. Method of Abstracting Primary Care Evaluation and Management Visits

Outpatient primary care workload visits were classified as evaluation and management (E&M) visits if any of the visit's diagnostic Current Procedural Terminology (CPT) codes matched the Berenson-Eggers Type of Service (BETOS) codes for "M1A Office Visits – New" or "M1B Office Visits – Established." Below is the full list of CPT codes corresponding to the M1A and M1B BETOS codes.

EM CPT Codes (BETOS in (M1a, M1b)

BETOS Code	CPT Code	CPT Name
M1A	0500F	INITIAL PRENATAL CARE VISIT
M1A	99201	OFFICE/OUTPATIENT VISIT NEW
M1A	99202	OFFICE/OUTPATIENT VISIT NEW
M1A	99203	OFFICE/OUTPATIENT VISIT NEW
M1A	99204	OFFICE/OUTPATIENT VISIT NEW
M1A	99205	OFFICE/OUTPATIENT VISIT NEW
M1A	99381	INIT PM E/M NEW PAT INF
M1A	99382	INIT PM E/M NEW PAT 1-4 YRS
M1A	99383	PREV VISIT NEW AGE 5-11
M1A	99384	PREV VISIT NEW AGE 12-17
M1A	99385	PREV VISIT NEW AGE 18-39
M1A	99386	PREV VISIT NEW AGE 40-64
M1A	99387	INIT PM E/M NEW PAT 65+ YRS
M1A	G0101	CA SCREEN;PELVIC/BREAST EXAM
M1A	G0245	INITIAL FOOT EXAM PT LOPS
M1A	G0248	DEMONSTRATE USE HOME INR MON
M1A	G0402	INITIAL PREVENTIVE EXAM
M1B	0502F	SUBSEQUENT PRENATAL CARE
M1B	0503F	POSTPARTUM CARE VISIT
M1B	1000F	TOBACCO USE ASSESSED
M1B	2000F	BLOOD PRESSURE MEASURE
M1B	95115	IMMUNOTHERAPY ONE INJECTION
M1B	95117	IMMUNOTHERAPY INJECTIONS
M1B	99058	OFFICE EMERGENCY CARE
M1B	99211	OFFICE/OUTPATIENT VISIT EST

© 2026 Staloff J et al. *JAMA Network Open*.

M1B	99212	OFFICE/OUTPATIENT VISIT EST
M1B	99213	OFFICE/OUTPATIENT VISIT EST
M1B	99214	OFFICE/OUTPATIENT VISIT EST
M1B	99215	OFFICE/OUTPATIENT VISIT EST
M1B	99354	PROLONGED SERVICE OFFICE
M1B	99355	PROLONGED SERVICE OFFICE
M1B	99366	TEAM CONF W/PAT BY HC PRO
M1B	99367	TEAM CONF W/O PAT BY PHYS
M1B	99391	PER PM REEVAL EST PAT INF
M1B	99392	PREV VISIT EST AGE 1-4
M1B	99393	PREV VISIT EST AGE 5-11
M1B	99394	PREV VISIT EST AGE 12-17
M1B	99395	PREV VISIT EST AGE 18-39
M1B	99396	PREV VISIT EST AGE 40-64
M1B	99397	PER PM REEVAL EST PAT 65+ YR
M1B	99401	PREVENTIVE COUNSELING INDIV
M1B	99402	PREVENTIVE COUNSELING INDIV
M1B	99403	PREVENTIVE COUNSELING INDIV
M1B	99404	PREVENTIVE COUNSELING INDIV
M1B	99411	PREVENTIVE COUNSELING GROUP
M1B	99412	PREVENTIVE COUNSELING GROUP
M1B	99420	HEALTH RISK ASSESSMENT TEST
M1B	99429	UNLISTED PREVENTIVE SERVICE
M1B	G0246	FOLLOWUP EVAL OF FOOT PT LOP
M1B	G0247	ROUTINE FOOTCARE PT W LOPS
M1B	G0250	MD INR TEST REVIE INTER MGMT
M1B	G0420	ED SVC CKD IND PER SESSION
M1B	G0421	ED SVC CKD GRP PER SESSION

eAppendix 2. Measure Names and Definitions[17]

Measure Name and Number	Numerator	Denominator
**Influenza Immunization**		
19-65 years (p29h_ec)*	Eligible patients receiving influenza immunizations from 7/1 to 6/30	Veterans ages 19-65
66+ years (p28h_ec)*	Eligible patients receiving influenza immunizations from 7/1 to 6/30	Veterans 66 and older
**Cardiovascular Care**		
Controlling High Blood Pressure (ihd53h_ec)*	Veterans with a Blood Pressure that is less than 140/90 mm Hg in the reporting year	Eligible Veterans with Hypertension
Statin therapy (Mod/High dose) for patients with cardiovascular disease (statn1_ec)*	Eligible male Veterans 21-75 years of age and eligible female Veterans age 40-75 at the end of the reporting period who:<br>-had at least one VA dispensing event for a moderate or high-intensity statin medication during the reporting period (year); OR<br><br>-Have an ACTIVE non-VA statin medication of moderate or high-intensity during the reporting period (year) and verified with one of the following national Health Factors: VA-STATIN NON-VA MED VERIFIED, STATIN RX DOSE REVIEWED AND UPDATED, OUTSIDE STATIN-MODERATE OR HIGH DOSE; OR<br>-Are on a non-VA statin of moderate or high-intensity with an updated medication Start Date or Documentation Date during reporting period (year)	Eligible male Veterans 21-75 years of age and eligible female Veterans 40-75 years of age discharged with Acute Myocardial Infarction (MI), Coronary Artery Bypass Graft (CABG), Percutaneous Coronary Intervention (PCI) or with documented ischemic vascular disease (IVD) within the past two years
Statin adherence for patients with cardiovascular disease (statn4_ec)	Veterans who have moderate or high intensity statin medication prescribed and dispensed by VHA for at least 80% of days in treatment period	Male Veterans ages 21-75 and female Veterans ages 40-75 on the last day of the reporting year with at least one fo the following during the reporting year or the year prior: MI or old MI; CABG; PCI; Other revascularization; IVD AND received a VA-dispensed statin medication of moderate or high intensity
**Behavioral Health Measures**		

© 2026 Staloff J et al. *JAMA Network Open*.

Screened annually for depression (mdd40)**	Eligible patients screened for depression annually using the "PHQ-2" or "PHQ-9" with item-wise recording of item responses and total score. PHQ-2 was the required tool on/after 1/1/21: Two item depression screen which has high sensitivity but poor specificity for depression, leading to false positive rates. While appropriate for case finding further evaluation is required to establish an accurate diagnosis. Maximum score is 6, and a positive score is 3 or more. PHQ-9 is an acceptable tool and will be accepted beginning FY23Q4. PHQ-9 Nine-item Patient Health Questionnaire is a validated self- or interviewer administered instrument that assesses symptoms and effects on functioning. In addition, it can be scored as a continuous measure to assess severity and monitor treatment response. Maximum score is 27 and positive score is 10 or more.	All patients eligible for depression screening
Tobacco Use- Screened for Use-NEXUS (Outpatient) (p7)**	Eligible patients who were screened annually for tobacco using the mandatory National Clinical Reminder for Tobacco Use	Patients seen in eligible NEXUS clinics
Tobacco Use Cessation – Discussed Cessation Strategies (Outpatient)(smg9)**	Patients using tobacco Every Day or Some Days where an "Acceptable Provider" discussed or offered information on behavioral counseling options to assist with quitting in the past year using the National Clinical Reminder for Tobacco use (NEXUS)	Patient seen in eligible NEXUS clinics who report using smoking or using tobacco Every Day or Some Days
Veterans Screened Annually for Alcohol Misuse – Outpatient (sa7)**	Veterans screened annually for alcohol misuse using the AUDIT-C with item-wise recording of item responses and a total score for the AUDIT-C is documented in the medical record	All Veterans eligible for alcohol misuse screen

* eQM Measure
** EPRP Measure

© 2026 Staloff J et al. *JAMA Network Open*.
