# Supplementary material for: Primary Care by Telehealth and Care Quality in the Veterans Health Administration
Source: JAMA Netw Open. 2026 Feb 17;9(2):e2559940. doi: 10.1001/jamanetworkopen.2025.59940 (PMC12914489; doi:10.1001/jamanetworkopen.2025.59940)
Supplement: Supplement 2. — Data Sharing Statement [file jamanetwopen-e2559940-s002.pdf]

Data Sharing Statement

Staloff. Primary Care by Telehealth and Care Quality in the Veterans Health Administration. *JAMA Netw Open*. Published February 17, 2026. doi:10.1001/jamanetworkopen.2025.59940

Data
Data available: No

Additional Information
Explanation for why data not available: Due to US Department of Veterans Affairs (VA) regulations and our ethics agreements, the analytic data used for this evaluation are not permitted to leave the VA firewall without a Data Use Agreement. VA data are made freely available to VA authorized researchers with an approved VA protocol. For more information, please visit https://www.virec.research.va.gov or contact the VA Information Resource Center at VIReC@va.gov
